# 

**Published:** 1840-12

**Authors:** 


### CONTENTS

OF NO. XII.

---

### ORIGINAL COMMUNICATIONS.

### ESSAYS AND CASES.

Art. I.—Remarks on Negro Consumption. By A. H. Buchanan, M. D., of Columbia, Tennessee - - - - - 405

Art. II.—Cases Illustrative of the Beneficial Effects of the Oil of Turpentine. By Dr. John Bennett, late of Newport, Kentucky. - - - - - - - - - 418

Art. III.—Cases of Gangrene of the Mouth. Reported to the Fairfield Medical Association, Ohio, by James White, M. D. Communicated for publication by the Secretary. - - - - - - - - - - 422

Art. IV.—A Case resembling Fungus Hæmatodes successfully treated. Reported to the Medical Society of Tennessee. By Samuel Henderson, M. D. - - - 433

### REVIEWS.

Art. V.—American Journal of the Medical Sciences. No. LI. Article XII, a Review of Gross's "Elements of Pathological Anatomy," under the signature of T. S. - - 435

SELECTIONS FROM AMERICAN AND FOREIGN JOURNALS.

On the Operation for Strabismus. By Professor DIEFFENBACH. - 475

ORIGINAL INTELLIGENCE.

To Readers and Correspondents. - - - - - - - 479
Medical Convention of Kentucky. - - - - - - 481
Medical Obituary. - - - - - - - - - 481
Medical and Physiological Commentaries. By MARTYN PAYNE, M. D., A. M. - - - - - - - - - - 484
An Introductory Lecture, by Professor Lindsly, of the Columbian College. November, 1840. - - - - - - - 484

